# Platinum: Reusing Constraint Solutions in Bounded Analysis of Relational Logic

**DOI:** 10.1007/978-3-030-45234-6_2

**Published:** 2020-03-13

**Authors:** Guolong Zheng, Hamid Bagheri, Gregg Rothermel, Jianghao Wang

**Affiliations:** 8 University of Paderborn, Paderborn, Germany; 9 ICREA, Open University of Catalonia, Barcelona, Spain; 10 Department of Computer Science and Engineering, University of Nebraska-Lincoln, Lincoln, NE USA; 11 Department of Computer Science, North Carolina State University, Raleigh, NC USA

## Abstract

Alloy is a lightweight specification language based on relational logic, with an analysis engine that relies on SAT solvers to automate bounded verification of specifications. In spite of its strengths, the reliance of the Alloy Analyzer on computationally heavy solvers means that it can take a significant amount of time to verify software properties, even within limited bounds. This challenge is exacerbated by the ever-evolving nature of complex software systems. This paper presents Platinum, a technique for efficient analysis of evolving Alloy specifications, that recognizes opportunities for constraint reduction and reuse of previously identified constraint solutions. The insight behind Platinum is that formula constraints recur often during the analysis of a single specification and across its revisions, and constraint solutions can be reused over sequences of analyses performed on evolving specifications. Our empirical results show that Platinum substantially reduces (by 66.4% on average) the analysis time required on specifications extracted from real-world software systems.

## 1 Introduction

The growing reliance of society on software and software-intensive systems drives a continued demand for increased software dependability. Software verification provides the highest degree of software assurance, with its strengths residing in the mathematical concepts that can be leveraged to prove correctness with respect to specific properties. Most notably, bounded verification techniques, such as Alloy [28], have recently received a great deal of attention in the software engineering community (e.g., [8, 9, 11, 13, 14, 16, 20, 26, 34, 35, 38, 43, 46, 48, 52, 54, 55, 61, 63, 66]), due to the strength of their automated, yet formally precise, analysis capabilities. The basic idea behind these techniques is to construct a formula that encodes the behavior of a system and examine it up to a user-specified bound. They thus enable analyses of partial models that represent key aspects of a system.

Bounded verification techniques often transform a software specification to be analyzed into a satisfiability problem, and delegate the task of solving this to a constraint solver. In the past decade, constraint solving technologies have made spectacular progress (e.g., [19, 22, 42]). Despite these advances, however, constraint solving continues to be a bottleneck in analyses that rely on it [58]. This is because the magnitude of formulas tends to increase exponentially with the size of the system being analyzed, making it impractical to employ constraint solving on complex systems. Further, despite the many optimizations applied to constraint solvers, they are still unable to detect many instances of subformula recurrence that are generated by Alloy.

The foregoing challenges are exacerbated when considering the ever-evolving nature of complex software systems and their corresponding specifications. Formal specifications are developed iteratively, and each iteration involves repeated runs of the analyzer for assessment of their semantics [31, 36]. In online analyses, where specifications are kept in sync with the evolving software and analyses are performed at runtime, the time required to verify the properties of software is of even greater significance. This calls for techniques that assist constraint solvers in dealing with large corpora of formulas, many of which contain tens of thousands of clauses.

In this paper, we introduce PLATINUM, an extension of the Alloy Analyzer that supports efficient analysis of evolving Alloy specifications, by recognizing opportunities for constraint reduction and reuse of previously identified constraint solutions. Unlike the Alloy Analyzer and its other variants, e.g., Aluminum [45], that dispose of prior results in response to changes in the system specification, PLATINUM stores solved constraints incrementally, and retrieves them when they are needed again within the analysis of the revised specification. PLATINUM further improves analysis efficiency by omitting redundant constraints from a specification before translating them into propositional formulas to be solved by expensive constraint solvers, thereby greatly reducing the required computational effort. Although techniques for storing the results of satisfiability checking and reusing them later have been considered in the context of symbolic execution [6, 7, 29, 49, 62], these techniques cannot be directly applied to Alloy due to the specifics of its core logic, which consolidates the quantifiers of first-order logic with the operators of the relational calculus [28]. (Section 5 provides details.)

We evaluate the performance of PLATINUM in several scenarios. First, we apply PLATINUM to several pairs of specifications in which the second contains a small but non-trivial set of changes relative to the first. Second, we apply PLATINUM to several sequences of specifications that model evolution scenarios. Our empirical results show that PLATINUM is able to support reuse of constraint solutions both within a single analysis run and across a sequence of analyses of evolving specifications, while achieving speed-up over the Alloy Analyzer. Third, we show that as the scope of the analysis increases, PLATINUM achieves even greater improvements. Fourth, we show that the overhead associated with PLATINUM is a fraction of that required by the Alloy Analyzer. Finally, we show that PLATINUM substantially reduces (by 66.4% on average) the analysis time required on specifications extracted from real-world software systems.

This paper makes the following contributions:

- *Efficient analysis of evolving relational logic specifications.* We present a novel approach to improve the bounded analysis of relational logic specifications by transforming constraints into more concise forms and enabling substantial reuse of solutions, which in turn substantially reduces analysis costs.
- *Tool implementation.* We implement PLATINUM as an extension to Alloy and its underlying relational logic analyzer, Kodkod [57]. We make PLATINUM available to the research and education community [5].
- *Empirical evaluation.* We evaluate PLATINUM in the context of Alloy specifications found in prior work and specifications automatically extracted from real-world systems, corroborating PLATINUM's ability to substantially outperform the Alloy Analyzer without sacrificing soundness or completeness.

## 2 Illustrative Example

To motivate this research and illustrate our approach, we provide a simple Alloy specification and describe the analysis process followed by the Alloy Analyzer and PLATINUM.

Consider snippets of the Alloy specification for a simple customer-order class diagram, shown in Listing 1.1 (adapted from [15]). Each Alloy specification consists of data types and formulas that define constraints over those data types. A signature (sig) paragraph introduces a basic data type and a set of its relations, called *field*s, accompanied by the type of each field. The running example defines seven signatures (Lines 2–21). The Customer class (Lines 2–7) has two attributes, customerID and customerName, that are assigned to the attrSet field of the Customer class. The id field specifies that customerID is the identifier of this class. The last two lines of the Customer signature specification indicate that Customer is not an abstract class and that it has no parent. Similarly, the code in Lines 10–15 represents the Order signature specification, and CustOrder (Lines 18–21) specifies an association relationship between Customer and Order.

```
1  // (a) a simple customer-order class diagram
2  one sig Customer extends Class{}{
3    attrSet = customerID +customerName
4    id=customerID
5    isAbstract = No
6    no parent
7  }
8  one sig customerID extends Integer{}
9  one sig customerName extends string{}
10 one sig Order extends Class{}{
11   attrSet = orderID + orderValue
12   id=orderID
13   isAbstract = No
14   no parent
15 }
16 one sig orderID extends Integer{}
17 one sig orderValue extends Real{}
18 one sig CustOrder extends Association{}{
19   src = Customer
20   dst = Order
21 }
22 fact associationMultiplicity{
23   one CustOrder.src and some CustOrder.dst
24 }
```

```
1  // (b) new constructs added to the revised specification
2  one sig PreferredCustomer extends Class{}{
3    attrSet = discount
4    one parent
5    parent in Customer
6    isAbstract = No
7    id=customerID
8  }
9  one sig discount extends Integer{}
```

Listing 1.1: (a) a specification describing a simple customer order class diagram; (b) new constructs added to a revised version of that specification.

Facts (fact) are formulas that take no arguments, and define constraints that each instance of a specification must satisfy, restricting the specification's solution space. The formulas can be further structured using predicates (pred) and functions (fun), which are parameterized formulas that can be invoked. The associationMultiplicity fact paragraph (Lines 22–24) states multiplicities of source and destination classes in the CustOrder association relationship.

To analyze such a relational specification, both the Alloy Analyzer and PLATINUM translate it into a corresponding finite relational model in a language called Kodkod [56]. Listing 1.2 shows a partial Kodkod translation of Listing 1.1(a). A specification in Kodkod's relational logic is a triple

```
{C1,O1}

Customer:  (1,1)::[{<C1>},{<C1>}]
Order:     (1,1)::[{<O1>},{<O1>}]
parent:    (0,4)::[{},
                  {<C1,C1>,<C1,O1>,<O1,C1>,<O1,O1>}]

(no Customer.parent) && (no Order.parent) ...
```

Listing 1.2: Kodkod representation of the Alloy specification of Listing 1.1 (partially elided for space and readability).

consisting of a universe of elements (a.k.a. *atoms*), a set of relation declarations including lower and upper bounds specified over the model's universe, and a relational formula in which the declared relations appear as free variables [56].

The first line of Listing 1.2 declares a universe of two uninterpreted atoms. (Due to space limitations, the listing omits some of the relations and atoms.) While in Kodkod all relations are untyped, in the interest of readability we assume an interpretation of atoms in which C1 represents a Customer element and O1 represents an Order element.

Lines 3–6 of Listing 1.2 declare relational variables. Similar to Alloy, formulas in Kodkod are constraints defined over relational variables. Whereas in Alloy these relational variables are separated into *signatures* that represent *unary* relations establishing a type system, and *fields* that represent non-unary relations, in Kodkod all relations are untyped, with no difference made between unary and non-unary variables.

Kodkod also allows scope to be specified from above and below each relational variable by two *relational constants*; these sets are called *upper* and *lower* bounds, respectively. In principle, a relational constant is a prespecified set of tuples drawn from a universe of atoms. Each relation in a specification solution must contain all tuples that appear in the lower bound, and no tuple that does not appear in the upper bound. That is, the upper bound represents the entire set of tuples that a relational variable may contain, and the lower bound represents a partial solution for a specification.

```
1 (!(v1|v2))&(!v2|!v1)&(!(v3|v4))&(!v4|!v3)
2
3 Slices:
4 (!(v1|v2))&(!v2|!v1)
5 (!(v3|v4))&(!v4|!v3)
6
7 Canonical form:
8 (!(1|2)&(!2|!1))
```

Listing 1.3: Excerpt of the boolean encoding for the Kodkod specification shown in Listing 1.2.

Consider the Customer declaration (Listing 1.2, Line 3). Both its upper and lower bounds contain just one atom, C1, given that it is defined as a singleton set in Listing 1.1. The upper bound for the variable $parent \subseteq Class \times Class$ (Lines 5–6) is a product of the upper bound set for its corresponding domain and co-domain relations, here $(Customer \cup Order) \rightarrow (Customer \cup Order)$, taking every combination of an element from both and concatenating them.

To transform such a finite relational model into a boolean logic formula, Kodkod renders each relation as a boolean matrix, in which any tuple in the upper bound of the given relation that is not in the lower bound maps to a unique boolean variable [56]. Relational constraints are then captured as boolean constraints over the translated boolean variables.

To render this idea concrete, consider the parent relation along with the next constraint defined over it (Listing 1.2, Lines 5–8). Each of the four tuples in the upper bound of the parent relation is allocated a fresh boolean variable (v1 to v4) in the boolean encoding. The relational constraint (no Customer.parent) && (no Order.parent) is then translated as a boolean constraint over those boolean variables, as shown in Listing 1.3, Line 1.

Expressions and constraints in relational specifications typically contain equivalent slices in their boolean representations. PLATINUM detects such semantically redundant slices by refining the specification in its boolean logic form into its essential, independently analyzable slices, and then rendering them in a canonical form. The boolean encoding of the constraints defined over the parent relation, for example, embodies two slices with equivalent but syntactically distinct formulas (Listing 1.3, Lines 4–5). Line 8 represents the result of restructuring the slices into a canonical form, suggesting that the two slices are in fact equivalent. The slicing technique we use to determine the sets of clauses, the satisfiability of which can be analyzed independent of other clauses in the formula, is presented in Section 3.

PLATINUM prevents redundant slices from being propagated to the CNF formula to be solved by the underlying SAT solver, substantially reducing computational effort. In the case of our example specification (Listing 1.1(a)), PLATINUM partitions the original relational specification into 30 slices, with only seven distinct canonical slices. As such, PLATINUM is faster at finding a solution instance, requiring 19 ms to do so compared to the 26 ms that the Alloy Analyzer requires to produce the first solution instance. The time required to compute the entire instance set also improves, from 6481 ms to 246 ms, in this simple example.

PLATINUM also reuses results produced for specification slices to further improve the analysis of evolving specifications. Consider Listing 1.1(b), for example, in which two new signature paragraphs are added, stating that the PreferredCustomer class inherits from the Customer class. Given the updated specification, PLATINUM reuses the results from the prior run and solves a smaller problem. Specifically, after slicing and canonicalizing the formula, the results for 29 slices, out of the total of 30 slices, are already available. As a result, PLATINUM requires only one millisecond to find the first solution for the revised specification, whereas the Alloy Analyzer requires 27 milliseconds to produce the first solution. PLATINUM also produces speed-ups in computing the whole solution space. In the case of this particular example, PLATINUM reduces the time required to produce the entire solution set from 768 milliseconds to two milliseconds.

## 3 Approach

Fig. 1 provides an architectural overview that shows how PLATINUM fits in with Alloy. As the figure shows (left), the Alloy Analyzer reads in an Alloy specification and translates it into a relational model, then passes that to Kodkod. Kodkod translates the relational model into a boolean formula, then to CNF, and passes the CNF to off-the-shelf SAT solvers to obtain a solution. Last, the Alloy interpreter translates the SAT result into a solution instance.

PLATINUM is inserted between Kodkod and the Alloy interpreter, as shown in the figure. At the highest level, PLATINUM takes in the boolean formula from Kodkod and outputs SAT results to the Alloy interpreter. The box at right shows the steps PLATINUM follows to do this. PLATINUM first decomposes the boolean formula into independent slices. Then, for each slice, PLATINUM canonicalizes it into a normalized format and searches the storage for a previously existing equivalent slice. If such a slice exists, the previous results will be reused. Otherwise, the slice is translated to CNF and assigned to an independent SAT solver for processing. Both the slice and the results of processing it

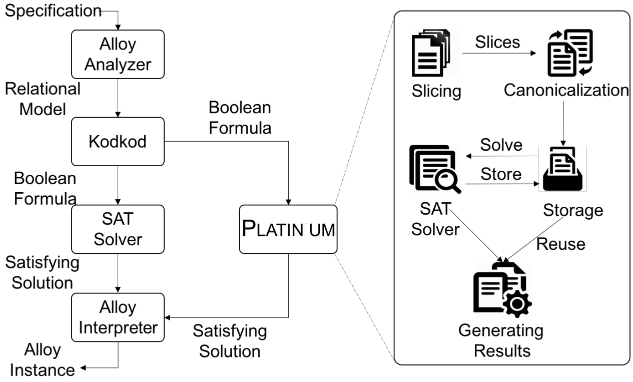

Fig. 1: Overview of Alloy and PLATINUM

are then stored. Finally, PLATINUM combines the results for each slice and passes them to the Alloy interpreter.

Next, we describe each step taken by PLATINUM in detail.

### 3.1 Slicing

In PLATINUM, the slicing operation takes in the boolean formula generated from Kodkod and decomposes it into a set of independently analyzable subformulas. Formally, given a boolean formula $\varphi$, slicing decomposes it into subformulas $\varphi_1, \varphi_2, ..., \varphi_n$, such that the following equations hold:

- $\varphi_1 \wedge \varphi_2 \wedge ... \wedge \varphi_n = \varphi$
- $var(\varphi_1) \cup var(\varphi_2) \cup ... \cup var(\varphi_n) = var(\varphi)$
- $var(\varphi_i) \cap var(\varphi_j) = \emptyset$, for each $\varphi_i$ and $\varphi_j$ where $i \neq j$
- $var(\varphi_i) \neq \emptyset, for\ i = 1, 2, ..., n$

where $var(\varphi)$ is the set of boolean variables of $\varphi$. Subformulas $\varphi_1$ to $\varphi_n$ can be solved independently. Thus, $\varphi$ is satisfiable if and only if each slice $\varphi_i$ is satisfiable individually.

**Algorithm 1** Slicing

```
Require: f: original Boolean Formula root
Ensure: Slices: Set of Independent Slices
procedure SLICE(f)
    Slices ← null
    for each variable v ∈ f do
        parent[v] ← v
        rank[v] ← v
    end for
    DECOMPOSE(f)
end procedure procedure DECOMPOSE(f)
    if f.operator = AND then
        for each subformula f_i ∈ f do
            DECOMPOSE(f_i)
        end for
    else
        UNION-FIND(f)
    end if
end procedure
```

A boolean formula can be sliced either logically (based on semantics) or algebraically (based on syntax). In the interest of efficiency, PLATINUM applies a syntactic slicing algorithm. There are two types of boolean formulas in Alloy: a propositional formula that Kodkod translates from the relational model and the conjunctive normal form generated from the propositional formula. PLATINUM applies slicing on the propositional formula level for two reasons. First, translating a propositional formula to CNF introduces many auxiliary variables [21]. For example, when the CustomerOrder specification in Section 2, with 81 variables in its propositional formula, is translated to a CNF formula containing 352 variables, 271 auxiliary variables are introduced. The explosion in the number of variables affects the performance of slicing and canonicalization. Second, in certain cases, auxiliary variables connect two independent formulas together. Given the boolean formula $v_1 \& v_2$, its CNF translation is $(v_1|!o)\&(v_2|!o)\&(!v_1|!v_2|o)$, where $o$ is the auxiliary variable. Even if $v_1$ and $v_2$ are independent formulas, in the CNF, $v_1$ and $v_2$ are dependent on each other.

Slicing can be viewed as identifying connected components in a graph, where the vertices of the graph are boolean variables and the edges of the graph represent two variables that appear within the same clause. Each slice is thus one connected component in the graph. The conventional way to proceed with this is to first build a graph for the entire boolean formula, and then run a depth-first-search (DFS) to identify each connected component [62]. For large specifications this can be both time and memory intensive. To improve performance, our algorithm applies a modified UNION-FIND algorithm [17], that traverses the boolean formula only once to identify connected components.

**Algorithm 2** Union-Find

```
procedure UNION-FIND(f)
    represent ← null
    for each variable v ∈ f do
        if v has been visited then
            if UnMeetState then
                represent ← FINDSLICE(v)
                add f to Slices[represent]
                change to MeetState
            else
                if FINDSLICE(v) != FINDSLICE(represent) then
                    UNIONSLICES(Slices[represent],Slices[v])
                end if
            end if
        else
            UNIONVARS(v, represent)
            v.visited ← TRUE
        end if
    end for
end procedure procedure UNIONVARS(v,represent)
    if represent is null then
        represent ← FINDSLICE(v)
    end if
    Parent[represent] ← FINDSLICE(v)
    Rank[represent] ← Rank[represent] + 1
end procedure procedure UNIONSLICES(represent,v)
    v ← FindSlice(v)
    if Rank[represent] ≤ Rank[v] then
        Slices[v].add(Slices[represent])
        Parent[represent] ← v
        Rank[v] ← Rank[represent] + Rank[v]
    else
        Slices[represent].add(Slices[v])
        Parent[v] ← represent
        Rank[represent] ← Rank[represent] + Rank[v]
    end if
end procedure procedure FINDSLICE(v)
    while v != Parent[v] do
        v ← Parent[v]
        Parent[v] ← Parent[Parent[v]]
    end whilereturn v
end procedure
```

Algorithm 1 outlines the slicing process. Given boolean formula root, the algorithm first initializes a data structure used by its subroutine (Lines 2–6). Each slice is identified by a representative, which is one variable within the slice. Array Parent is used to find the representative variable. Array Rank is used to construct a balanced parent array. Array Slices maps a representative variable to its corresponding slice; its size equals the number of slices. The algorithm then calls subroutine DECOMPOSE to decompose the root formula.

DECOMPOSE recursively partitions a boolean formula f into subformulas in such a way that the conjunction of all subformulas equals f, and each subformula cannot be decomposed into smaller subformulas.

The UNION-FIND procedure (Algorithm 2) takes a decomposed subformula and finds a slice to which it belongs. The basic idea behind the algorithm is that each slice is represented by one variable. UNION-FIND has two basic operators: *UNION* and *FIND*. If *UNION* operates on two slices, it joins them into one slice (Lines 27–38). If *UNION* operates on two variables, it assigns one variable to be the parent of the other (Lines 20–26). The *FINDSLICE* operation determines the representative variable for the slice – the variable to which the input variable belongs. It does so by traversing the Parent array until it finds one variable $v_p$ whose parent is itself, i.e., parent[$v_p$] = $v_p$. All variables along this path belong to the same slice and are represented by $v_p$.

The input boolean formula has two states: UnMeetState, which indicates that *f* does not belong to any slice yet, and MeetState, which indicates that *f* belongs to some slice that is represented by *represent*. For each variable *v* of the input boolean formula *f*, UnMeetState first obtains the representative variable for *v* (which could be itself if *v* does not belong to any slice yet). If *v* has not been visited, the algorithm unions *v* and the representative variable of the subformula (Lines 20–26). Otherwise, if *v* has been visited (i.e., it belongs to some slice), and if *f* is in UnMeetState, then the algorithm adds *f* to the slice represented by *represent*. Finally, if *f* is in MeetState, this means that *f* belongs to one slice and *v* belongs to another and these need to be joined together (Lines 27–38).

## 3.2 Canonicalization

The time complexity of the UNION-FIND algorithm is near linear [17]. Without this improvement and using the conventional DFS-based approach taken by Green [62] among others, in one case in our empirical study, a few minutes were required to produce independently analyzable slices. Using our algorithm, this time was reduced to about 10 milliseconds – an order of magnitude speedup. This speedup occurs for the following reason. A graph is needed to start the DFS. The graph contains information about which variable belongs to which clause and which clause contains which variables, and a map-like data structure is needed to store this information. When the number of variables becomes huge—typically hundreds of thousands in formulas produced for Alloy specifications of real-world systems—it is time and memory consuming to obtain this information and store it. It is also time consuming to retrieve the graph information during the DFS. Our UNION-FIND based algorithm, in contrast, requires information only on the node's parents, and this can be placed in a static array that requires only linear time to store and retrieve.

**Algorithm 3** Canonicalization

```
Require: f : boolean formulas
Ensure: f′ : canonical boolean formula
procedure CANONICALIZE(f)
    varSet ← var of f
    varSet ← sort(varSet)
    for i in 0 to varSet.length do
        labelMap.add(varSet[i].label, i)
        varSet[i].label ← i
    end for
    L ← varSet.length
    for each subformula sf ∈ f do
        RENAME(sf)
    end for
    f′ ← f
end procedure procedure RENAME(f)
    for each subformula sf ∈ f do
        L ← RENAME(sf)
    end for
    f.label ← ++L
    returnL
end procedure
```

The slices produced by the prior step are passed to this step, which transforms each slice into a canonical format in order to capture the syntactic equivalence between different slices. For a slice $\varphi$, where $\varphi = \varphi_1 \wedge \varphi_2 \wedge \ldots \wedge \varphi_n$, canonicalization generates one boolean formula $\varphi'$, such that $\varphi' = \varphi'_1 \wedge \varphi'_2 \wedge \ldots \wedge \varphi'_n$, where $\varphi'$ is the canonical format of $\varphi$. The canonical form of the formulas is constructed by renaming variables and formula labels. Algorithm 3 outlines this transformation.

Canonicalization first renames each boolean variable based on its weight (Lines 2–7). For each variable $v \in V$, where $V = var(\varphi_1) \cup var(\varphi_2) \cup \ldots \cup var(\varphi_n)$, the weight of $v$ is calculated as the sum of the number of its occurrences and the number of operators applied on $v$ in all of the subformulas. To improve the performance of this step, the weight for each variable is collected during the slicing phase; then, $V$ is sorted based on variable weight. If two variables have the same weight, their original labels are used to sort them. Each variable is then renamed to their index in the sorted array. The mapping relations from canonical variables to original variables for each slice are stored in *labelMap* for use in assembling the solution for the original boolean formula. Next, the label for each formula is renamed (Lines 8–20). The purpose of this step is to maintain consistency with variables when translating to CNF. The labels of formulas are used as auxiliary variables when they are translated to CNF.

### 3.3 Storing and Reuse

After slicing and canonicalization have been completed, each boolean formula is decomposed into several independent formulas. For each canonicalized boolean formula, PLATINUM checks its hash code in storage. If there is a hit, this boolean formula is already solved, and the result will then be retrieved. If not, the boolean formula will be translated into CNF and solved by the SAT solver independently. The result will then be stored.

After solving all slices, using the *labelMap* (Algorithm 3) that maps canonical variables to original variables, PLATINUM obtains the solution for the original boolean formula and passes it to Alloy to generate a solution instance.

## 4 Empirical Study

We empirically evaluated the performance of PLATINUM in relation to the following research questions:

**RQ1:** How does the performance of PLATINUM compare to the performance of existing approaches on specifications that have undergone relatively small amounts of change?

**RQ2:** How does the performance of PLATINUM compare to the performance of existing approaches on specifications that have gone through several successive rounds of evolution?

**RQ3:** How does the performance of PLATINUM compare to the performance of existing approaches on specifications that have run against higher scopes?

**RQ4:** What is the overhead of PLATINUM in restructuring a relational logic formula into its canonical form?

**RQ5:** How does the performance of PLATINUM compare to the performance of Alloy Analyzer in practice on specifications automatically extracted from real-world applications?

### 4.1 Objects of Analysis

Our objects of analysis are specifications drawn from a variety of sources and problem domains. These specifications vary widely in terms of size and complexity. Table 1 lists the specifications that we use, with statistics on their size in terms of the numbers of relations in their underlying logic. Note that this number, in turn, represents the sum of the numbers of signatures and fields, as both are indeed translated into relations in the underlying relational logic.

Table 1: Objects of Analysis

| Specification | # Rels |
|---|---|
| Ecommerce | 70 |
| Decider | 47 |
| CSOS | 64 |
| Wordpress | 54 |
| Andr. Bundle 1 | 665 |
| Andr. Bundle 2 | 558 |
| Andr. Bundle 3 | 485 |
| Andr. Bundle 4 | 569 |
| Andr. Bundle 5 | 501 |
| Andr. Bundle 6 | 456 |

Ecommerce is a model, adopted from Lau and Czarnecki [30], that represents a common architecture for open-source and commercial E-commerce systems. Decider [15] is a model of a system to support design space exploration. CSOS is a model of a cyber-social operating system meant to help coordinate people and tasks. WordPress is an object model obtained by reverse engineering an open-source blog system [3]. Finally, the last six rows of the table correspond to six large specifications intended for the analysis of security properties in the context of the Android platform. Each consists of a bundle of Android applications installed on a mobile device for detecting security vulnerabilities that may arise due to inter-application communication, adopted from [12].

For the first four objects of analysis, we do not have access to actual, modified versions of their Alloy specifications, and even if we did, there would not likely be enough versions to provide data sufficient to support quantitative analyses. Thus, instead, we used a mutation-based approach to create modified versions of the specifications. We used edit operations for Alloy specifications [10] and incorporated into the MuAlloy mutation system [64] to derive a list of mutation operators. Table 2 provides a list of these mutation operators, together with short descriptions.

To investigate RQ1 we wished to apply our mutation operators to create 30 modified versions of each of our objects of study. Because prior work by Li et al. [31] showed that users tend to modify Alloy specifications incrementally by small amounts, we chose to create versions of our object specifications by mutating between one and 10% of the relations in the specifications. Given object specification $S$, for each modified specification $S'$ of $S$ to be created, we randomly chose a number $N$ in this range;

Table 2: Mutation Operators

| | Description |
|---|---|
| ADS | Add a new signature |
| DLS | Delete a signature without children |
| CSM | Change the signature multiplicity,[3] i.e., to set, one, lone or some (one that is different from the multiplicity defined in the original specification) |
| ABS | Make an abstract signature non-abstract or vice versa |
| MOV | Move a sub-signature to a new parent signature |
| ADF | Add a new field to a signature declaration |
| DLF | Delete a field from a signature declaration |
| CFM | Change a multiplicity constraint in a field declaration |

$N$ denotes the number of mutations to apply to $S$. We then began randomly choosing relations $L$ in $S'$, then randomly choosing a mutation operator $M$ applicable to $L$, and applied $M$ to $S'$. We did not allow a given $L$ to be utilized more than once in this process. Following each operator application, we ran Alloy on the current version of $S'$ to ensure that it is a valid specification. We repeated this process until $N$ mutations had been inserted into $S'$. Ultimately, this process produced 30 modified versions of each object specification, wherein each version contained a randomly selected number of randomly selected mutations – a number no greater than 10% of the number of relations in the original specification.

To investigate RQ2 we used a similar process; however, in this case our goal was to "evolve" each object specification $S$ iteratively. Given the original version $S$, we created a successor version $S_1$ by repeating the process of inserting a randomly selected number of randomly selected mutations (again, a number no greater than 10% of the number of relations in $S$). However, our next iteration applied this same process to $S_1$ (which now contains a number of mutations) to produce a version $S_2$ that potentially contains more mutations. Here, we say "potentially" because we did not place any restrictions on the re-use of mutation operators or mutation locations in subsequent versions $S_k$ of $S$; thus, conceivably, a mutation could be "undone" in a subsequent version. We repeated this process 30 times on each specification, thereby obtaining a sequence of specifications that have evolved iteratively.

It is common for users of bounded verification techniques such as Alloy to increase the scope of the analysis, in order to obtain greater confidence in the validity of the specification. As the scope of analysis increases, the space of cases that must be examined expands dramatically. To investigate RQ3, we increased the scope of analysis on each of our object specifications. Note that the only change in the specification between two successive runs of the analyzer in this case was the scope of analysis.

To investigate RQ4 we used the dataset created for RQ1. To investigate RQ5, we created six different app bundles, each containing 20 Android apps drawn from public app repositories such as Google Play [2]. We then used the COVERT tool [1] to automatically extract Alloy specifications from the app bundles. Given an original bundle $B$, we created a successor version $B'$ by adding a new app or removing an existing app (randomly selected) to/from the given bundle. The specifications automatically derived from app bundles tend to evolve as apps are added to, or removed from, the bundles. The resulting app bundles thus provide us with an ideal suite of evolving specifications that can be used for our evaluation. We repeated this process 30 times on each of the app bundles to produce 30 modified versions of each bundle specification.

## 4.2 Variables and Measures

**Independent Variables** As independent variables we wished to utilize PLATINUM, as well as baseline techniques representing state of the art approaches capable of performing the same function as PLATINUM.

We consider the Alloy Analyzer (version 4.2) as a baseline technique to compare against PLATINUM. The other potential baseline technique is Green [62], an optimization technique that, during symbolic execution, memoizes and reuses the results of satisfiability checking. The current implementation of Green, however, has two fundamental problems in the context of this study. First, while Green supports the use of Integer and Real variables in expressions, it does not support the use of boolean variables, which are widely used in the context of Alloy's relational logic. We were able to work around this challenge, however, by modeling boolean variables as Green's Integers and limiting their size to zero and one – an approach suggested by Green's developers. A more insidious problem, however, is that the Green framework does not currently support constraints with the disjunction operator. Because Alloy specifications are in relational logic, native support for the disjunction operator is essential to effectively analyze such specifications. This issue has been reported to the Green repository [4], and we have been in contact with the authors about it; however, to date, the issue has not been resolved and there are no workarounds for it. Thus, we were ultimately unable to use Green as a baseline technique.

Additional independent variables used were (b) the size of specifications in terms of relations in the relational logic, (c) the number of mutation operations, (d) the type of mutation operations, and (e) the scope of the analysis.

**Dependent Variables** We measure several dependent variables. The first variable, analysis time, tracks performance directly. Here, we measure the wall clock time required to run (1) a complete Alloy analysis and (2) a complete PLATINUM analysis on each specification considered. The second variable is the number of unique, independently analyzable slices produced by PLATINUM for each specification under analysis. The third variable is the number of slices for which solutions are already available for each specification under analysis. Finally, the fourth variable is the size of the generated CNF formulas that must be solved by the underlying SAT solver. In the last case, we record the number of CNF variables and clauses produced by each of the two techniques when translating high-level Alloy specifications into SAT formulas.

### 4.3 Study Operation

For RQ1 and RQ3, for each of our specification pairs, we applied the Alloy Analyzer and PLATINUM, measuring the time required by each approach, and the number of variables and clauses at the SAT level produced by each tool.

For RQ2, for each of our specification sequences, we applied both the Alloy Analyzer and PLATINUM to each pair of successive specifications in the sequence, measuring, for each iteration, the time required by each approach, the size of the SAT formula produced by each tool, and the number of slices reused across sequences.

For RQ4, for each of our specification pairs, we applied PLATINUM, measuring the time required for formula restructuring, including the slicing and canonicalization steps.

Finally, for RQ5, for each of the specification pairs extracted from app bundles, we applied both the Alloy Analyzer and PLATINUM, measuring the time required by each approach.

All of our runs of the Alloy Analyzer and PLATINUM were conducted on an 8-core 2.0 GHz AMD Opteron 6128 system with 40 GB of memory. Both techniques leveraged SAT4J as the SAT solver across the entire study to keep extraneous variables constant.

### 4.4 Threats to Validity

*External validity* threats concern the generalization of our findings. We have studied ten sets of Alloy specifications and cannot claim that they are representative of all such specifications. Additionally, our modified specifications for the first four objects of analysis were created via a mutation approach, and while this allows us to obtain large amounts of data, these objects may not directly represent modified specifications that exist in practice. To reduce this threat and help determine whether our results may generalize, we conducted additional studies using real-world software systems, where both the Alloy specifications and their revisions are automatically extracted from evolving bundles of real Android apps. Finally, different versions of the Alloy Analyzer may leverage different translation algorithms to CNF, and this may affect the execution time of the analyzer. To reduce this threat we used the latest stable release of the Alloy Analyzer, Alloy Analyzer 4.2, for all runs collected in the study.

*Construct validity* threats concern our metrics and measures; we are aware of no such threats in this case.

### 4.5 Results for RQ1 (Small Changes)

We first assess the effectiveness of PLATINUM with respect to the incremental changes derived from our first four object specifications. The boxplots in Fig. 2 depict the size of the generated CNF formulas, given as the number of variables (Fig. 2a) and clauses (Fig. 2b) across mutations for each object of study. The results show that in comparison to the Alloy Analyzer, PLATINUM's translation of relational logic specifications results in much smaller and simpler SAT formulas, and the numbers of CNF variables and clauses generated by PLATINUM were smaller than the numbers generated by Alloy. Specifically, in the analyses of the CSOS, Decider, Ecommerce, and Wordpress specifications, the numbers of variables and the numbers of clauses in the formulas produced by PLATINUM on average were 4.5/2.6/5.1/3.5 and 2.1/1.4/2.0/1.7 times lower, respectively, than the numbers in the formulas produced by the Alloy Analyzer. This is because already analyzed slices do

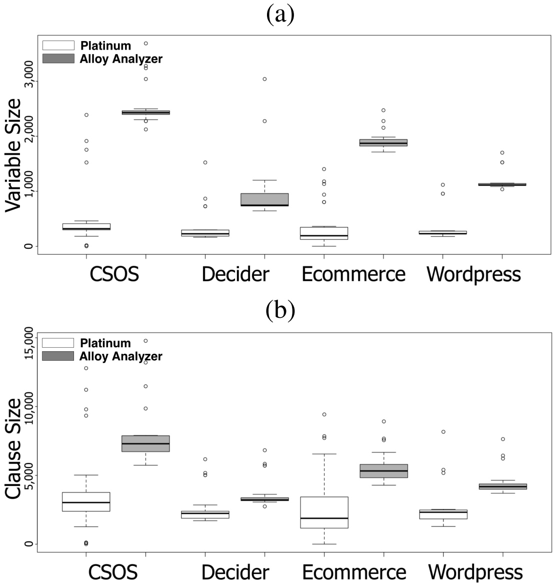

Fig. 2: Sizes of generated CNF formulas in terms of the number of (a) variables and (b) clauses produced by the Alloy Analyzer and PLATINUM across mutations for each object of study.

not need to be translated into SAT formulas, thus reducing the sizes of the generated CNF formulas.

Table 3 shows the results of a comparison of the average analysis times required by the Alloy Analyzer and PLATINUM across the four objects of study. On average, PLATINUM exhibited a 67.16% improvement over the Alloy Analyzer, with the average improvement across objects of study ranging from 16.82% to 82.31%.

Table 3: Performance Statistics

| | Alloy Analysis Time (S) | PLATINUM Analysis Time (S) | % Improvement |
|---|---|---|---|
| Ecommerce | 280.92 | 49.69 | 82.31% |
| CSOS | 120.64 | 56.71 | 52.99% |
| Wordpress | 57.19 | 47.57 | 16.82% |
| Decider | 27.38 | 5.69 | 79.21% |
| Average | 121.53 | 39.91 | 67.16% |

These results demonstrate the potential effectiveness of our optimization technique, because in every case, the analysis time required by PLATINUM to find solution instances of mutated specifications was less than that required by the state of the art analysis techniques.

### 4.6 Results for RQ2 (Successive Changes)

To assess the effectiveness of PLATINUM in accelerating analysis in successive runs on evolving specifications we use two performance metrics: *time ratio* (TR) and *variable ratio* (VR). We define the time ratio as $\frac{t_P}{t_A}$, where $t_P$ is the analysis time taken by PLATINUM and $t_A$ is the analysis time taken by the Alloy Analyzer. Intuitively, lower values of TR imply greater speedup. A TR of 0.5, for example, indicates that PLATINUM is two times faster than the Alloy analysis of the same specification, whereas a TR of 0.1 indicates that PLATINUM is 10 times faster. Similarly, we define the variable ratio as $\frac{var_P}{var_A}$, where $var_P$ is the number of variables in a SAT formula produced by PLATINUM and $var_A$ is the number of variables in a SAT formula produced by the Alloy Analyzer for the same specification. Again, lower values of VR imply that there are fewer variables in a

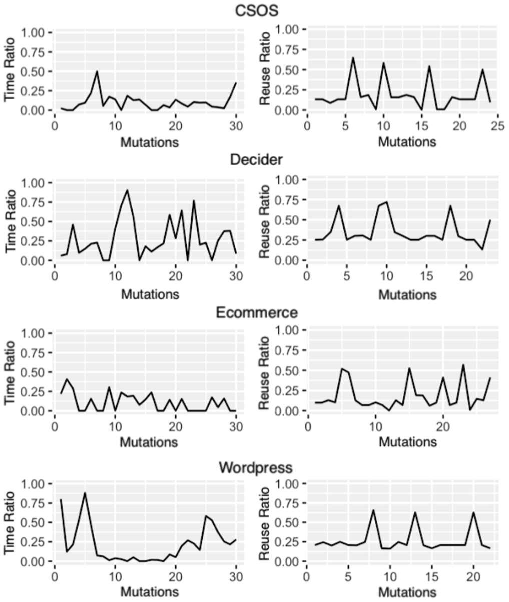

Fig. 3: Speedup and reuse during successive mutation analyses across subject domains. The left column represents scatter plots of time ratios (Analysis time taken by PLATINUM / Analysis time taken by Alloy), and the right column represents scatter plots of reuse ratios (#Variables in the SAT formula transformed by PLATINUM / #Variables in the SAT formula transformed by the Alloy Analyzer) across systems.

formula generated by Platinum than in a formula generated by the Alloy Analyzer. We started Platinum with an empty cache, and then analyzed each mutation in turn, continually populating the cache.

Fig. 3 presents a pair of diagrams for each of the four object specifications, demonstrating speedup and reuse during successive mutation analyses. The left column represents scatter plots of time ratios across subject domains, and the right column represents scatter plots of variable ratios. All four sets of experiments exhibit similar behavior: in every case, and for every revision, the analysis time taken by Platinum is less than that of using the Alloy Analyzer (values of TR are always less than 1), and the number of variables in formulas generated by Platinum is significantly less than those generated by the Alloy Analyzer. The speedup, however, varies for different mutations. Variation across mutations is expected, given that the size and complexity of the mutations produced in successive runs differ greatly. In a few cases, the values for TR jump. Investigation of the data shows that this occurred because the mutations present in those cases contained several new slices not yet observed, which in turn reduced the amount of reuse. Despite these few cases, the empirical results suggest that significant speedup was possible in all cases.

### 4.7 Results for RQ3 (Scope Changes)

Alloy's analysis is exhaustive, yet bounded, up to a user-specified scope on the size of the domains. In cases in which the analyzer fails to produce a solution that satisfies specification constraints within a given scope, a solution may be found in a larger scope. In practice, Alloy users often conduct consecutive analysis runs of specifications, applying small increases in the analysis scope, in the hopes of gaining further confidence in their results. It has been shown that 17.6% of consecutive Alloy analyses differ only in terms of their analysis scopes [31].

To examine how our optimization approach responds to increases in analysis scope, for each specification, we gradually increased the scope of the analysis. We set the initial scope for the analysis of each specification to the scope that had already been specified by its original modeler, reasoning that whoever had developed and analyzed the specification is most likely the best judge of the scope that is needed. The initial scopes for the CSOS, Decider, Ecommerce, and Wordpress specifications were 51, 27, 50, and 32, respectively. We started Platinum with an empty cache for the analysis of each specification, and gradually populated it as the analysis scope increased.

Table 4 shows the time ratios (TRs) measured as the analysis scope increased for each of the objects of study. Recall that lower values for TR indicate that greater acceleration was achieved by our optimization technique. The data shows that overall as scope increased, TR tended to decrease. For example, for the Ecommerce system, the lowest value for TR occurred when the scope increased to five, resulting in a 1 / 0.031 = 32 fold analysis speed acceleration.

Table 4: Analysis Time Improvements Over Increasing Sizes of Analysis Scope

| Scope increase | +1 | +2 | +3 | +4 | +5 |
|---|---|---|---|---|---|
| CSOS | 0.765 | 0.122 | 0.098 | 0.118 | 0.035 |
| Decider | 0.393 | 0.036 | 0.038 | 0.023 | 0.034 |
| Ecommerce | 0.727 | 0.234 | 0.413 | 0.049 | 0.031 |
| Wordpress | 0.486 | 0.107 | 0.079 | 0.053 | 0.079 |

### 4.8 Results for RQ4 (Overhead)

We next evaluate the performance of PLATINUM's formula restructuring analysis. Table 5 shows the time required to restructure relational logic formulas into their canonical forms. The first column represents the time spent decomposing formulas into independent slices, and the second column represents the time spent canonicalizing them into normalized formats.

Table 5: Analysis Times With Respect to Overhead Incurred Due to Restructuring of Formulas

| | Slicing Time(ms) | Canon Time(ms) | %overhead |
|---|---|---|---|
| CSOS | 7 | 268 | 0.36% |
| Decider | 3 | 41 | 0.63% |
| Ecommerce | 10 | 116 | 1.01% |
| Wordpress | 5 | 138 | 2.44% |
| Average | 6.25 | 140.75 | 1.11% |

As the data shows, the analysis time overhead incurred by these two steps is 1.11% on average, and no greater than 2.44% in any case. This is negligible, particularly when compared to the analysis time overhead incurred by the Alloy Analyzer (cf. Table 3). While the restructuring steps introduce little overhead, they substantially enable reuse of slice solutions, which in turn greatly reduces analysis costs.

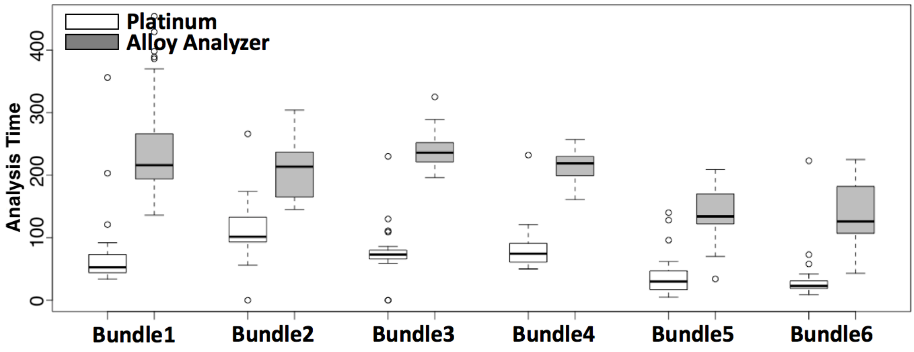

Fig. 4: Analysis times for the Alloy Analyzer and PLATINUM across specifications from real-world Android apps.

### 4.9 Results for RQ5 (Real-World Specifications)

Finally, to assess the improvements one could expect in practice using PLATINUM, we used Alloy specifications that were automatically extracted from real-world software systems and evolved versions thereof, as described in Section 4.1. Fig. 4 shows the results of a comparison of the analysis time required by each of the two techniques as boxplots across the six bundle specifications. As the results show, PLATINUM exhibited a 66.4% improvement, on average, over the Alloy Analyzer; the average improvement across app bundles ranged from 44.2% to 78.4%, indicating relative stability across bundles. These results further confirm those obtained through our mutation-based experiments, corroborating the effectiveness of PLATINUM in improving the analysis time required by the Alloy Analyzer to find solution instances of revised specifications.

## 5 Related Work

The literature contains a large body of research related to ours. Here, we provide an overview of the most notable and closely related work and examine it in the light of our research.

The widespread use of Alloy has prompted a number of extensions to the language and its underlying automated analyzer [10, 23, 24, 25, 27, 32, 37, 39, 40, 41, 45, 53, 58, 59]. Among these, Titanium [10] presents an exploration space reduction strategy that narrows the space of values to be explored by an underlying constraint solver. This approach, however, requires an entire solution set to be produced for the original specification, to determine tighter bounds for certain relations in the revised specification. Our work differs primarily in its emphasis on reducing constraints into a more concise form at the level of relational logic abstractions, which in turn allows for substantial reuse of analysis efforts in subsequent analyses. Research efforts on bound adjustment and solution reuse are complementary in that, in spite of the adjustments made to the analysis bounds, the solver still needs to solve for the shared constraints.

Uzuncaova and Khurshid [60] partition a specification into base and derived slices, in which a solution to the base slice can be extended to produce a solution for the entire specification. PLATINUM is fundamentally different from this work in that the problem addressed by Uzuncaova and Khurshid assumes a fixed specification and does not consider specification evolution. Further, their approach does not eliminate the need to solve shared, canonicalized constraints across analyses.

Rosner et al. [51] present a technique, Ranger, that leverages a linear ordering of the solution space to support parallel analysis of first-order logic specifications. While the linear ordering enables partitioning of the solution space into ranges, there is no clear way in which it can be extended with incremental analysis capabilities, which are crucial for effective analysis of evolving specifications.

Several techniques attempt to explore specification instances derived from Alloy's relational logic constraints [18, 33, 44, 45, 56]. Macedo et al. [33] examine scenario explorations in the context of relational logic. Aluminum [45] extends the Alloy Analyzer to generate minimal specification instances. Both of these efforts focus primarily on the order in which solutions are produced, as opposed to facilitating analysis of evolving specifications, which is our goal. Montaghami and Rayside [39] extend the Alloy language to explicitly support partial modeling. Their work, however, does not consider evolving specifications. In fact, it is widely recognized that efficient techniques for analyzing Alloy specifications are needed [58]. To the best of our knowledge, however, no prior research has attempted to reduce the need to call a solver to improve the efficiency of the analysis of evolving Alloy specifications.

The technique most closely related to ours is Green [62]; this technique has been the subject of several more recent papers [6, 7, 29, 47, 49, 50, 65], that improve on its algorithm. As noted in Section 1, Green and its offshoots also rely on back-end constraint solving engines. In contrast to all of this prior work, the problem we address in this paper involves supporting the evolutionary analysis of relational logic. Among other things, this requires the development of both original slicing and canonicalization approaches appropriate for models specified in Alloy's relational logic. Moreover, neither Green's slicer nor its canonicalizer take into account the disjunction operator [4]. While the lack of support for the disjunction operator might be allowable in the context of symbolic execution, that support is essential in the context of first-order logic to allow an approach to effectively recognize opportunities for constraint reduction and reuse. Further, while most of the prior techniques use a classic lexicographic ordering of the variables before transforming each slice into a canonical format, PLATINUM leverages a reverse shortlex order, in which the variables are first sorted by their weight and then sorted lexicographically. This choice improves the identification of syntactic equivalence between different slices. To the best of our knowledge, PLATINUM is the first technique for evolutionary analysis of relational logic specification that operates without requiring an entire solution set for the original specification.

## 6 Conclusions

We have presented PLATINUM, a novel extension to the Alloy Analyzer that substantially improves the process of analyzing evolving Alloy specifications. Our approach proceeds by storing solved constraints incrementally, and reusing them within subsequent analysis of a revised specification. It also omits redundant constraints from specifications before translating them into formulas that will be sent to constraint solvers. Our evaluation of PLATINUM shows that it is able to support substantial reuse of constraint solutions across analyses of evolving specifications. Our empirical results show significant speedup over the Alloy Analyzer in various scenarios. Our evaluation also shows that as the scope of analysis increases, PLATINUM achieves even further improvements, and that the overhead associated with the approach is negligible. Finally, our evaluation shows that PLATINUM continues to result in savings on specifications extracted from real-world software systems.

Our future work involves extending the optimization ideas presented here to leverage domain-specific knowledge. Specifically, we intend to explore the possibility of driving the automated discovery of domain-specific optimizations, wherein each system of interest can have bounded verification tailored to its specific characteristics. While such optimizations historically have arisen from the insights of a few dozen experts in software verification, we envision a bounded speculative analysis to identify how operations permissible within a certain domain may impact the exploration space of bounded analyses, thereby facilitating efficient analysis of specifications in a given domain.

## Acknowledgment

We would like to thank the anonymous reviewers for their valuable comments. This work was supported in part by awards CCF-1755890 and CCF-1618132 from the National Science Foundation.
